# Lessons learned evaluating the baby friendly spaces program for south Sudanese refugees in Gambella, Ethiopia: strengthening research and programmatic partnerships to address maternal and child health and psychosocial needs in humanitarian emergencies

**DOI:** 10.1186/s13031-020-00299-5

**Published:** 2020-07-25

**Authors:** M. E. Lasater, G. M. Woldeyes, K. Le Roch, X. Phan, A. Solomon-Osborne, S. M. Murray

**Affiliations:** 1 Department of Mental Health, Johns Hopkins Bloomberg School of Public Health, Baltimore, USA; 2 Mental Health and Care Practices, Gender and Protection, Action Against Hunger, Addis Ababa/Gambella, Ethiopia; 3 Mental Health and Care Practices, Gender and Protection, Action contre la Faim, 14-16 boulevard Douaumont, 75017 Paris, France

**Keywords:** Breastfeeding, Child care practices, Psychosocial support, Humanitarian emergencies, Refugees, Ethiopia, Process evaluation

## Abstract

**Background:**

During humanitarian crises, women and children are particularly vulnerable to morbidity and mortality. To address this problem, integrated child health interventions that include support for the well-being of mothers must be adapted and assessed in humanitarian settings. Baby Friendly Spaces (BFS) is a holistic program that aims to improve the health and wellbeing of pregnant and lactating women and their children under two years of age by providing psychosocial support and enhancing positive infant and young child-care practices. Using a mixed-methods, pre-post design, this study explored ways to strengthen the implementation and acceptability of the BFS program, and assess outcomes associated with participation among South Sudanese mothers and their children living in the Nguenyyiel refugee camp in Gambella, Ethiopia.

**Discussion:**

A stronger evidence-base for integrated maternal and child health interventions, like BFS, in humanitarian emergencies is needed, but effectively conducting this type of research in unstable settings means encountering and working through myriad challenges. In this paper we discuss lessons learned while implementing this study, including, challenges related to ongoing local political and tribal conflicts and extreme conditions; implementation of a new digital data monitoring system; staff capacity building and turnover; and measurement were encountered. Strategies to mitigate such challenges included hiring and training new staff members. Regular weekly skype calls were held between Action Against Hunger Paris headquarters, the Action Against Hunger team in Gambella and Johns Hopkins’ academic partners to follow study implementation progress and troubleshoot any emerging issues. Staff capacity building strategies included holding brief and focused trainings continuously throughout the study for both new and current staff members. Lastly, we engaged local Nuer staff members to help ensure study measures and interview questions were understandable among study participants.

**Conclusions:**

Research focused on strengthening program implementation is critically important for improving maternal and child health in humanitarian emergencies. Research in such settings demands critical problem-solving skills, strong supervision systems, flexibility in timeline and logistics, and tailor-made training for program and research staff members and context- adapted strategies for retaining existing staff.

## Background

### Humanitarian context

South Sudan seceded from Sudan in 2011 to become the world’s youngest nation [1]. While independence brought a brief respite from over three decades of civil war, accusations of an attempted coup in December 2013 by the country’s new president, Salva Kiir, once again provoked widespread conflict that has been responsible for an estimated 400,000 deaths [2]. This conflict, combined with drought and an economic crisis, exacerbated widespread poverty and precipitated famine [2]. Despite a peace deal being signed in 2018 [3], humanitarian efforts to support the South Sudanese have been hampered by poor or non-existent infrastructure, extremely limited availability of government services, sporadic tribal conflicts, and flooding [1]. Currently, approximately 4.3 million South Sudanese are displaced as a result of violence, food insecurity, and natural disaster [4].

Approximately 420,000 South Sudanese have relocated to one of six refugee camps in bordering areas of Ethiopia [1]. Nguenyyiel, the newest and largest Ethiopian camp, opened in 2016 near Gambella town and less than 100 km from the South Sudan border [5]. Built to accommodate 60,000 South Sudanese [5], as of April 2019, 74,095 refugees were living in Nguenyyiel, 91% of whom were of Nuer ethnicity [6], and over two thirds of whom were under 18 years of age [7]. In Nguenyyiel, there is a 29.5% prevalence rate of global acute malnutrition in children under age 5, almost two times greater than emergency threshold [8, 9]. Additionally, a recent survey in Nguenyyiel found that 45% of respondents reported mental health symptoms that are either sometimes or often severe enough to cause daily functioning impairment [5].

### Research study

In response to the urgent health and psychosocial needs of mothers and their children in emergency settings, Action Against Hunger (France) developed the Baby Friendly Spaces (BFS) program. BFS delivers evidence-based care practices for infant and young children [10, 11] (e.g. breastfeeding and nutrition counseling, child play and stimulation, parenting skills) to strengthen mothers’ skills; and, psychosocial support (e.g. group discussions, stress management skills, psychoeducation) to enhance mothers’ well-being and internal resources to care for their children [12, 13]. Action Against Hunger has been working in the Gambella refugee camps since July 2011, and between October 2016 and June 2019, about 19,500 women and their children have benefited from BFS services. All activities have been adapted for the Nuer population to ensure cultural relevance. From October 2018 to March 2019, we conducted a mixed-methods formative process evaluation of the BFS program among South Sudanese mothers of young children in the Nguenyyiel refugee camp to better understand program implementation and to assess if BFS participation is related to improved child and maternal health outcomes (See Table 1 for research aims and questions). Data were drawn from three sources: (1) an embedded qualitative study with BFS program participants, non-participants, and BFS staff; (2) a prospective quantitative assessment administered to women enrolling in BFS before engagement in any program activities and again two months later; and (3) routine program monitoring and evaluation data.

**Table 1:** Overview of Study Aims and Research Questions

Aims (A)	Research Questions (RQ)
*A1*. Explore the uptake, acceptability and participation of women in the Baby Friendly Spaces (BFS) program.	*RQ1.* What are the perceived needs of South Sudanese mothers of young children (under age 2) in the BFS catchment area? *RQ2.* What are the characteristics of women who attend the BFS program, and each activity within the BFS program? *RQ3.* What activities are the most acceptable and useful to women? What activities are missing or not useful? *RQ4.* What are the reasons for program drop out or choosing to not-participate, and how do these women differ from those in the program?
*A2.* Explore the needs and experiences of staff delivering the BFS program.	*RQ1.* What are the staff’s experiences of delivering the BFS program (recruitment, content and monitoring and process evaluation), including challenges and solutions generated? *RQ2.* What are the needs of BFS staff to be supported in delivering the program effectively? *RQ3.* To what extent is the program being delivered with fidelity?
*A3.* Assess whether and how participation in BFS activities is associated with improved maternal and child outcomes.	*RQ1.* What are the overall patterns of health and wellbeing among women who enroll in BFS? *RQ2.* How do these outcomes vary by participation in activities and/or participant characteristics (demographics, baseline problems, etc.)?

Women were eligible for study participation if they were living in the Nguenyyiel refugee camp, aged 18 or older, currently breastfeeding a child under the age of 2, and fluent in Nuer or English. BFS staff members were eligible for study participation if they were age 18 or older and had been working in Nguenyyiel refugee camp, with the BFS program for at least one month. All study participants gave oral informed consent and ethical approval was obtained by the Institutional Review Boards at Johns Hopkins University and Jimma University in Ethiopia.

### Qualitative data collection

Semi-structured, in-depth interviews (IDIs) were conducted by two trained Nuer research assistants among 36 mothers including mothers who completed the BFS program (*n* = 12), mothers who dropped out (n = 12), and mothers who never participated (n = 12). The interviews focused on understanding the psychosocial needs of mothers, BFS program experiences, and barriers to participation. IDIs with BFS staff members (*n* = 8) explored program delivery challenges. Data were inductively coded and analyzed using a thematic analysis approach.

### Quantitative data collection

To quantitatively assess the health trajectories of women and their children in the BFS program, 201 mothers were recruited during program enrollment. Prior to enrolling participants, the assessment instrument was translated into Nuer and independently back translated into English to ensure semantic equivalency. The instrument included basic demographic items and multiple measures to assess behavioral and health outcomes of interest (see Table 2). Eleven locally trained psychosocial assistants administered the survey to women prior to participation in any BFS activities (baseline) and again two months later (endline). Data are being analyzed to examine any changes in the outcomes of interest and to see if variation in participation in the intervention (duration, extent, types of activities) predicted change in outcomes.

**Table 2:** Measures included in the quantitative assessment of participation in the BFS program

Outcome	Measure	Description
Maternal functional impairment	World Health Organization Disability Assessment Schedule (WHODAS 2.0)	12-item measure using a 5-point Likert scale asking respondents to indicate the level of difficultly they have due to health conditions over the past two-weeks. 3 additional items ask respondents to indicate the number of days 1) difficulties were present, 2) difficulties prevented you from engaging in your daily activities, and 3) days you had to cut back on usual activities.
General psychosocial distress	Kessler psychological distress scale-6 item (K-6)	Six-item measure using a 7-point Likert scale asking respondents about experiences of distress over the past two-weeks.
Depression symptoms*	Patient Health Questionnaire (PHQ-9)	Nine-item measure using a 4-point Likert scale, asking respondents to indicate the number of days over the past two-weeks they had experienced each depression symptom
Post-traumatic stress symptoms	PTSD Checklist (PCL-6)	Six-item measure using a 5-point Likert scale, asking respondents to indicate how much they have been bothered by each problem in the past two weeks.
Mother-child interactions		Seven-item measure that is used to rate four minutes of unstructured play between the mother and child.
Quality of Breastfeeding practices*	WHO BREAST Feed Observation form	An observation checklist assessing signs of a successful breastfeeding and signs of possible difficulties across six domains (body position, responses, emotional bonding, anatomy, suckling and time spent suckling).
Child health and growth	MUAC, illness inventory, WAZ/HAZ/WHZ	Mid-upper arm circumference, illness symptoms over the past two weeks (fever, diarrhea, respiratory difficulties, loss of appetite, skin problems, oedema, vomiting), WAZ (underweight for age), HAZ (stunting- low height for age), WHZ (wasting- low weight for height).

*Primary outcome; all other outcomes are secondary

## Discussion

### Scientific importance of this research

The health of women and children are adversely affected during humanitarian emergencies, placing them at heightened risk of morbidity and mortality [14, 15], particularly in contexts with pre-existing widespread poverty and disease [16–18]. Children under age two are particularly vulnerable when exposed to armed conflict due to being at greater risk during this critical developmental period of contracting infectious diseases, with potential long-term adverse effects on their nutritional and developmental trajectories, and a higher likelihood of death associated with incident illness [8, 19]. In non-conflict settings, maternal factors such as depression, breastfeeding practices, and child attachment are demonstrated risk factors for child health and development [20, 21]. Maternal depression has been associated with suboptimal rates of child immunization, well-child hospital visits, exclusive breastfeeding, and with higher rates of child diarrheal and febrile illnesses [22]; and has negative impacts on child cognitive, motor, and socio-emotional development [10, 23, 24]. Moreover, mounting epidemiological evidence from humanitarian settings highlights the importance of maternal mental health, mother-child attachment [25] and nutrition [26] for maternal and child mortality and morbidity.

As stated in the Lancet Series on Maternal and Child Nutrition (2013), while maternal mental health has a demonstrated association with child growth, the wellbeing of mothers is still too rarely taken into consideration in global health programming [20, 21, 26]. This is also true of health services in humanitarian emergencies, which rarely consider the interrelationship of the health of mothers and their babies through integrated, sustained, evidence-based postpartum care. Given the lack of rigorous evaluation of integrated maternal and neonatal health interventions in humanitarian settings [27], we felt it was imperative to evaluate the BFS program to ensure that the humanitarian response is efficient and appropriate when needs are great and resources are scarce, and that programs remain accountable to refugee beneficiary populations. Though changes have been observed in breastfeeding practices in Baby tents in Haiti [28], to date, a rigorous evaluation of BFS has not been conducted. This mixed methods process evaluation was designed as a first step towards an effectiveness evaluation to understand program acceptability and impact, and to strengthen monitoring and evaluation systems to better understand how the program is implemented. In addition, we sought to identify and address challenges in researching the program in a setting characterized by instability. While we expect the results of this study to be generalizable to other humanitarian emergency settings in eastern and central Africa, the extent to which the program needs to be tailored to reflect the unique cultural and environmental context of other emergencies is something we recommend for future study.

### Challenges to research

#### Ongoing conflict and extreme conditions

Frequent political and tribal conflicts took place in the Gambella region during our study period, creating a highly volatile and unpredictable environment. Following the large influx of Nuer communities from South Sudan beginning in 2016, political and economic tensions escalated between the Nuer and Anuak, leading to large-scale violent demonstrations in Gambella town and the refugee camps, and cycles of retributive attacks between groups that at times resulted in multiple fatalities [5]. Interclan violence between Nuer refugees living in the camp would also occur periodically, causing the road to Nguenyyiel to become unsafe and camp access to be restricted, particularly for Nuer staff members. Families also relocated to other zones within the camp or to the surrounding host communities in search of safety during conflict, leading to both program drop-out and difficultly locating women for follow-up data collection. On occasion, all humanitarian operations in the camp were suspended prioritizing staff safety.

Extreme heat also posed several challenges related to data collection. The Nguenyyiel refugee camp is very large in area, requiring some women to walk 30 min to reach the BFS center. During the hot season in Gambella, temperatures can reach above 40 degrees Celsius (104 F). Participants voiced complaints and discomfort related to the heat and concerns about exposing their young children to these conditions. In response to both conflict and heat, many programs beneficiaries restricted their movement within the camp, limiting the number of mothers who could safely reach the center to participate in BFS .

In part because of sporadic conflict in the camp, women were often consumed with efforts to meet basic needs. While UNHCR reports that each refugee in the camp has access to 10 l of potable water daily, water had to be trucked into the camp. Water shortages frequently occurred during outbreaks of violence when trucks could not access the camp to refill water stations [7]. During these times, infectious disease outbreaks were commonplace due to a lack of clean water. Women at times were unavailable at follow-up because they were seeking medical care for their children, sometimes outside of the camp. Prolongments in the monthly food distribution due to conflict in the camps also affected mothers’ ability to attend follow-up assessments. Moreover, recurrent exposure to stress and trauma, and potentially limited access to food rations and water during periods of instability could have confounded the relationship between BFS participation and the outcome variables of interest (maternal depression, child health and development, and quality of breastfeeding practices). Without a control group, it is difficult to assess to what extent these environmental factors potentiated changes in outcomes, yet there are ethical challenges to randomizing support in this setting where need is so severe.

#### Implementation of new data monitoring system

We (the research team and Action Against Hunger program staff), encountered multiple challenges introducing a new data management information system (DMIS) during the study to collect more rigorous program data, as well as data for this study. While using tablets to collect data electronically reduced data entry burdens and enabled complex data to be more readily available in theory, limited and slow internet access in Gambella made uploading data difficult. Additionally, it was difficult to procure needed materials locally (specifically barcode stickers for beneficiary tracking cards), and our qualitative findings suggested that losing the card led mothers to skip BFS activities. Staff also had limited prior exposure to smartphone-tablet applications and there was a lack of experienced information technology staff available locally to support the data management application during times of difficulty.

#### Staff capacity, disruption and turnover

During the initial stage of the study, research assistants needed individualized supervision to understand the study questionnaire and improve their interview skills due to limited prior experience with research. Additionally, BFS program supervisors at three of four sites did not speak Nuer, which limited their ability to provide supervisory support while Nuer research assistant conducted interviews in real time. Computer skills, particularly typing skills were limited among our qualitative research assistants, which made it impossible to transcribe interviews word for word in a feasible timeframe.

In addition to staffing disruptions caused by the conflict as described above, staff retention was affected by numerous international humanitarian aid actors competing for a limited number of trained Nuer/ English speaking staff members. For example, building the capacity of staff through high quality quantitative and qualitative data collection trainings opened the doors to staff recruitment by other NGOs offering better incomes. Waiting periods related to obtaining ethical approval and restricted camp access due to conflict created delays between initial staff training and research study implementation. This created more opportunity for turnover and may have affected staff motivation to participate in the research.

#### Conducting quantitative assessments in populations with limited educational opportunity

With one of the effects of living in a context of long-term instability being restricted access to education, challenges were also encountered related to participant literacy levels and familiarity with rating systems. Women often faced difficulties understanding questions, especially those related to mental health. Many women also found it difficult to understand and select between response options. While this challenge did not prevent women from providing responses, it did add time to survey administration.

### Research strategies

Several strategies were undertaken to mitigate each of the challenges we encountered during the study, as detailed in Table 3. Overall, a strong, efficient and well-organized partnership was established between Action Against Hunger headquarters, Action Against Hunger Ethiopia, and scientific partners from Johns Hopkins University. Moreover, throughout the research period we engaged local actors, including, United Nations High Commissioner for Refugees (UNHCR) and Administration for Refugee and Returnee Affairs (ARRA), who reviewed and authorized the research protocol in the camp. These relationships, developed at the beginning of the project, could be drawn upon during the course or research and will serve as means of dissemination at the conclusion of the research. Due to numerous challenges and to mitigate their impact on the project, availability and flexibility of the different actors involved in the project was necessary for maximizing adjustment strategies.

**Table 3:** Research challenges, solutions and key lessons learned

Research Challenges	Solutions	Key Lessons learned
*Ongoing conflict and extreme conditions* • Denied or restricted access to Nguenyyiel, particularly among Nuer staff members • Disruptions in BFS service provision, data collection and monitoring activities • Restricted movement in the camp, relocation in the camp or to the host community among study participants • Extreme heat • Recurrent exposure to stress and trauma among study participants	• Hire new staff and transfer staff members from neighboring camps to help deliver BFS services • Amend research protocol to allow for data collection in the homes of participants • Conduct community outreach activities to identify the new addresses of study participants after relocating	• Prioritizing and ensuring the safety and health of participants and BFS staff members requires critical problem-solving skills and flexibility to achieve the research objectives and minimize loss to follow-up.
*Implementation of a new data monitoring system* • Limited and slow internet access • Difficulty and delays uploading data and duplicate data entries • Limited exposure to smart-phone- tablet applications • Lack of local experienced information technology staff	• Perform data uploads at night or at the office of partner organizations with stronger internet connection • Hold regular skype calls with Action Against Hunger headquarters and use of Team Viewer (remote desktop software)	• Establish and troubleshoot new data and monitoring systems prior to conducting research.
*Staff capacity, disruption and turnover* • Need for individualized support among research assistants • Limited computer skills among qualitative research assistants • Staff turnover due to competitive employment packages from other humanitarian actors in the camp	• Hold remote trainings for new hires and current staff at the Action Against Hunger Gambella office • Allocate ample time for research assistants to practice administering the questionnaire and review all data collected with the respective research assistants to check for errors and identify solutions to any challenges • Conduct IDIs in pairs with one research assistant and expanded ID notes while listening to the audio following the interview • Conduct continuous recruitment of BFS Staff • Incentivize staff members with contract extensions • Weekly skype calls among the study team	• Allocate ample time to staff training and capacity building, and frame trainings as a career enhancing opportunity • Communicate challenges related to study implementation and delays with staff members • Include transcription skills in qualitative trainings
*Quantitative assessment in populations with limited education opportunities* • Illiteracy of study participants led to difficulty understanding questions and selecting response options	• Conduct independent translation and back translation of Nuer measures to make them more understandable among participants. • Update Nuer terminology and phrasing with assistance of Nuer staff members • Use flash cards with pictorial representations to aid participant understanding of response categories	• Work with local staff to help ensure the understandability and appropriateness of terminology and phrasing of questionnaire items.

#### Ongoing conflict and extreme conditions

In order to keep BFS program and research activities running as much as possible during periods of conflict, non-Nuer staff members were transferred from other camps in the region to address human resource needs at Nguenyyiel with the assistance of translators. We amended our research protocol to allow for data collection at participants’ homes so they would not have to travel during intense heat and to minimize research participation time given the amount of time they needed to spend meeting basic needs. However, we received ethical approval for these changes after we had completed data collection and thus did not implement the requested changes to our research protocol. We also allocated significant staff time to contact women at follow-up, particularly those who had moved during periods of conflict. Staff members not only called participants but went door-to-door to identify the new addresses of participants within the camp. We also consulted with the refugee counsel in the camp and key community members to help us locate women for their follow-up assessments.

#### Implementation of new data monitoring system

To address challenges related to the DMIS, direct support was provided from Action Against Hunger headquarters via regular skype calls, in addition to a DMIS training with members of the research team prior to data collection. Staff often uploaded data at night, when internet usage was lower and speeds were faster. On occasion, data was uploaded at the offices of partner international non-governmental organizations who had a stronger internet connection. Ultimately, after data collection ended, the use of this system was discontinued for ongoing program activities. Based on this experience, we highly recommend establishing new data and monitoring systems prior to the beginning of research in coordination with local partners’ and their expressed needs and dedicating one staff member to coordinate the training of the data management system, its use, and troubleshooting.

#### Staff capacity, disruption and turnover

In Gambella, allocating time for staff capacity building was a key factor for a successful project, especially given turnover and the limited research experience for most BFS staff. Importantly, while limited prior research experience led to some challenges, the staff’s language skills, knowledge of the local cultures, and ability to provide feedback about comprehension and relevance of the research project was essential to the conduct of rigorous, quality research. Prior to data collection, the research assistants participated in in-person research methods trainings with the research team, and practiced questionnaire administering with each other until they felt comfortable asking the questions and following skip patterns (which we minimized as much as possible). We also led focused trainings utilizing an integrated mentorship model to support a ‘learning while doing’ process, to ensure optimal data quality checks during data collection and entry. Additionally, at the end of each day of data collection the study coordinator would review all data with the respective research assistants to check for data errors and to address any challenges encountered during the course of data collection. The study team also had weekly calls with the study coordinator to review study progress, and to address any challenges encountered in the field. To address transcription challenges, research assistants conducted qualitative interviews in pairs, with one conducting the interview and the other taking detailed notes. Following the interviews, research assistants expanded upon their interview notes while listening to the audio and transcribed key participant quotes.

In addition, remote trainings that focused on data collection methods for new staff or refresher trainings for existing staff took place at the Action Against Hunger office in Gambella (outside of the camp) with the study coordinator (GM) and the first author (ML) via skype to make efficient use of staff time when camp access was restricted. Action Against Hunger received two no-cost extensions from the study funder and extended the contracts of existing staff as much as possible to ensure continuity of data collection and provision of BFS activities during the research period. Non-Nuer staff members delivering BFS at other camps in Gambella helped deliver BFS services and oversee program monitoring when faced with staffing shortages. The study coordinator provided direct supervision of all research assistants and scheduled weekly visits to each BFS site to oversee data collection. Based on these experiences, we highly encourage researchers to plan for multiple ways to provide refresher trainings or training of new staff, create standard operating procedures for supervision and oversight of research teams, and consider the need for a study coordinator to have both strong data management and interpersonal skills, from the beginning of research projects.

#### Adapting quantitative assessments to the target population

Despite the limited literacy of the population and difficulty translating some terminology, we selected mental health and psychosocial measures that had been successfully piloted and used in another study among South Sudanese refugees [29]. We conducted an independent back translation of the Nuer measures, reconciling differences between the original and translated English, in an effort to make the measures more accurate, understandable and culturally relevant to the target audience. Significant time was also devoted within the quantitative data collection training with Nuer staff toward discussing and updating terminology and phrasing as necessary, but less time was spent on how to guide participants in using ordinal response options or alternative strategies. We did create and use flash cards with pictorial representations to aid participant comprehension of response categories, but would recommend greater attention to training data collectors on how to help women who struggle with response categories, such as repeating response categories. In addition, we recommend the use of cognitive interviewing [30] to better understand how women respond to these types of items and alternatives that researchers can use to better quantify the responses of women with limited opportunities for education or exposure to this type of response style.

## Conclusions

Formative research focused on strengthening program implementation and monitoring and evaluation activities is critical for improving maternal and child health outcomes in humanitarian emergencies. Despite numerous challenges, research in humanitarian emergencies, such as ours, is critically important and possible, requiring planning and efficient problem solving that is only possible through strong research to practice partnerships. Throughout the study period we had to be flexible with the location of data collection activities, data management, recruitment, duties, and training of staff members, and the research timeline in order for activities to remain ongoing during periods of insecurity. Planning ahead for how to incorporate this flexibility is key to research success in humanitarian settings. Assessing programs like BFS is essential not only to ensure an efficient and appropriate humanitarian response, but also for developing systems and knowledge to support program implementation and overcome challenges, ultimately readying the program for evaluation in a comparative trial and effective dissemination of key findings to improve policy and practice.

## Data Availability

N/A

## Abbreviations

ARRA: Administration for Refugee and Returnee Affairs
BFS: Baby Friendly Spaces
DMIS: Data management information system
HAZ: Height-for-age z-score
IDI: In-depth interview
K6: Kessler Psychological Distress Scale
MUAC: Mid-upper arm circumference
PCL-6: Post-Traumatic Checklist- 6 Item Civilian Version
PHQ-9: Patient Health Questionnaire 9
RCT: Randomized controlled trial
UNHCR: United Nations High Commissioner for Refugees
WAZ: Weight-for-age z-score
WHO: World Health Organization
WHODAS: World Health Organization Disability Assessment Schedule
WHZ: Weight-for-height z-score
